# Nuclear accumulation of symplekin promotes cellular proliferation and dedifferentiation in an ERK1/2-dependent manner

**DOI:** 10.1038/s41598-017-04005-z

**Published:** 2017-06-19

**Authors:** Chen Zhang, Hai-Lei Mao, Yi Cao

**Affiliations:** 1 Laboratory of Molecular and Experimental Pathology, Kunming Institute of Zoology, Chinese Academy of Sciences, Kunming, China; 2 Kunming College of Life Science, University of Chinese Academy of Sciences, Kunming, China; 3 Department of Anesthesiology and Critical Care Medicine, Zhongshan Hospital, Fudan University, Shanghai, China

## Abstract

Symplekin is a multifunctional protein that localizes to both tight junctions and the nucleus in polarized epithelial cells, with confirmed roles in mRNA maturation, transcriptional modulation and tight-junction assembly. However, the mechanisms governing its subcellular distribution and related functions remain unclear. In this study, we found that symplekin primarily localizes to the nuclei of cultured dedifferentiated colorectal cancer cells, and nuclear symplekin showed higher phosphorylation and binding affinity with YBX3 than its membrane fraction. Moreover, the accumulation of nuclear symplekin promoted cell proliferation and dedifferentiation as well as β-catenin transactivation *in vitro*. Nuclear symplekin acts as a transcriptional co-activator for the expression of many cell cycle-related genes. Furthermore, extracellular signal-regulated kinase (ERK) phosphorylated symplekin at T1257 to facilitate its nuclear accumulation upon epidermal growth factor (EGF) stimulation. Meanwhile, reduction of total symplekin also induced certain epithelial-mesenchymal transition features in HT-29 cells. Taken together, our results confirm the coordinated roles of symplekin in cell junctions and gene transcription, which are related to its subcellular localization. The significance of nuclear symplekin in tumorigenesis is also highlighted, and ERK-dependent phosphorylation represents a mechanism for its subcellular sorting.

## Introduction

Symplekin is expressed in a wide range of cell types and participates in cytoplasmic mRNA polyadenylation^1^, cell proliferation^2^, differentiation^3^, mitosis^4^ and tumorigenesis^5^. Previously, we confirmed the role of symplekin in cell tight-junction (TJ) assembly and polarity maintenance^6^. Among peripheral TJ proteins, Zonula Occludin-1 (ZO-1), Y-box transcriptional factor 3 (YBX3, also known as CSDA, DBPA or ZONAB) and symplekin have been reported to form functional protein complexes and to shuttle between the junctional plaques and the nucleus^2, 6, 7^. In addition to polarized epithelial cells, symplekin localizes exclusively to the nucleus of tight-junctionless cells, emphasizing its vital roles in the nucleus^8^. However, the underlying mechanism, including the translocation of symplekin, is poorly understood. By comparing nuclear and extra-nuclear symplekin, we observed that nuclear symplekin exhibited increased phosphorylation in the present study. Post-translational modifications such as phosphorylation, glycosylation, and ubiquitylation have emerged as dynamic and essential regulators for target proteins sorting and relocalization to participate in various cellular events^9^. The phosphorylation of several junctional components, such as ZO-1, occludin and β-catenin, has also been shown to determine their subcellular distrbutions^10–12^. We found that extracellular epidermal growth factor (EGF) signals induced the phosphorylation of symplekin on specific residues, followed by nuclear translocation, with nuclear symplekin serving as a trans-activator to promote cell proliferation through the transcriptional modulation of several cell cycle-related genes via interactions with nuclear factor YBX3.

Epithelial TJs are highly dynamic intercellular structures that play multiple fundamental roles in organisms, such as supporting tissue organization, maintaining cell polarity and regulating paracellular semi-permeability^13, 14^. Along with adherence junctions (AJs) and desmosomes, TJs can form intact junctional complexes to maintain epithelial integrity^15^. Apart from their structural roles, the proteins that constitute TJs and AJs, e.g., ZO-1, β-catenin, p-120 catenin, etc., have been verified to participate in diverse signaling pathways and modulate various cellular events^12, 16, 17^. In the current work, we further reveal that tight junction-associated cytoplasmic symplekin is essential for the stability of epithelial junctional complexes. Coupled with its nuclear features, our findings help provide a comprehensive understanding of the multiple roles of symplekin in diverse cellular processes as a function of its subcellular distribution.

## Results

### Membrane symplekin translocates to the nucleus in dedifferentiated cells

**Figure 1:**
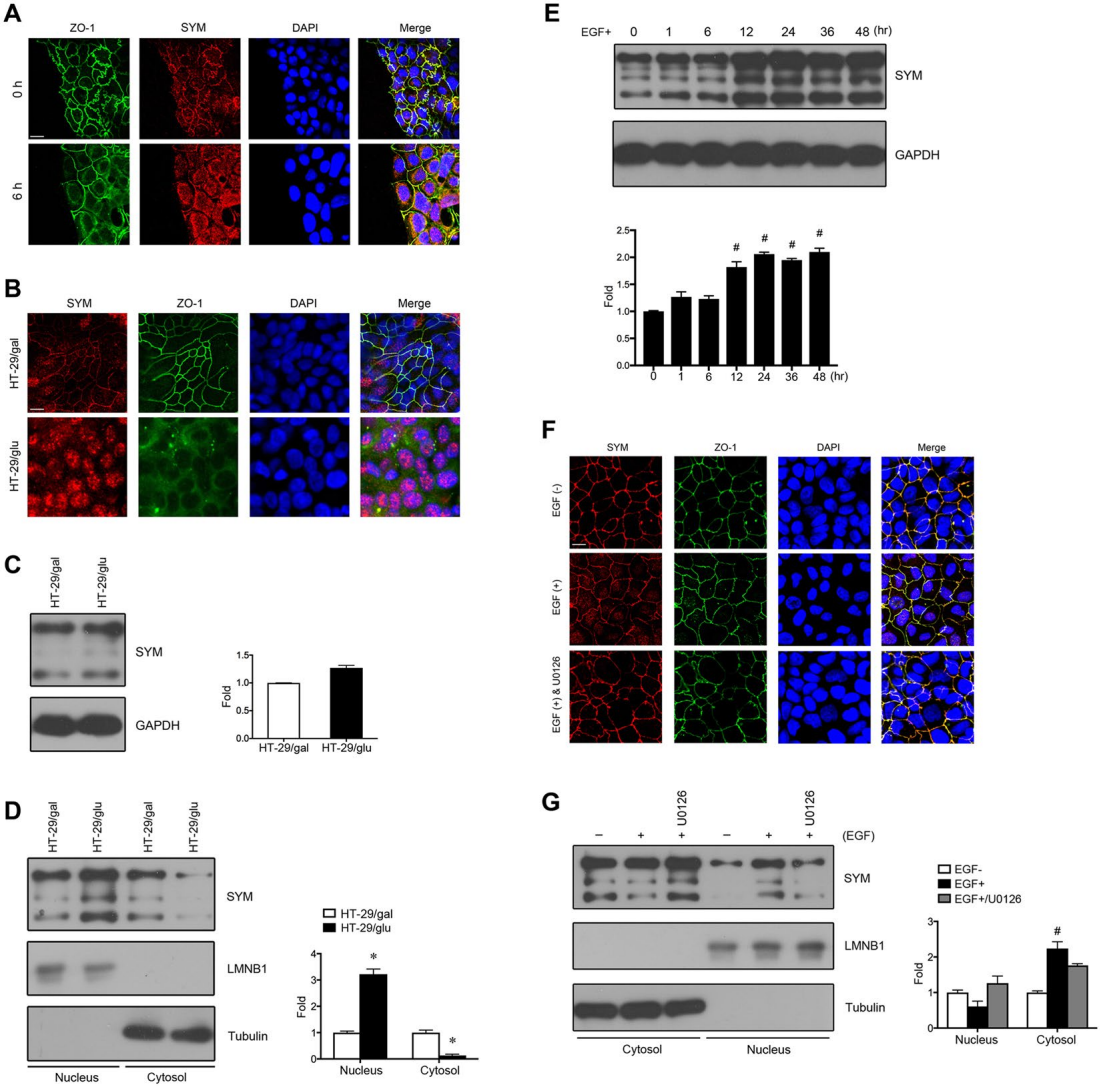
Expression and distribution of symplekin in dedifferentiated cells. (**A**) A confluent Caco-2 cell monolayer was scratched with a 1-ml pipette tip. Symplekin (SYM) and a tight junction marker (ZO-1) were immuno-stained at 0 hr and 6 hr after wound healing. Nuclei were stained with DAPI (blue). (**B**) Symplekin and ZO-1 staining in HT-29 cells cultured in glucose-free medium supplemented with galactose (HT-29/gal) or glucose (HT-29/glu). (**C**) Representative WB bands and relative densitometric quantification of total symplekin expression in HT-29/gal and HT-29/glu cells. (**D**) Representative WB bands of cytoplasmic/membrane (Cytosol) and nuclear (Nucleus) distribution of symplekin in HT-29/gal and HT-29/glu cells. Cytoplasmic tubulin and nuclear lamin B1 (LMNB1) were used as controls for quantification analysis, respectively. (**E**) Symplekin expression levels in starved Caco-2 cells treated with EGF for different time periods shown by bands of WB and relative quantitative graphs. (**F**) The localization of symplekin and ZO-1 in Caco-2 cells with (+) or without (−) EGF treatment. Cells were pre-treated with U0126 (40 μM) for 30 min before EGF stimulation. (**G**) Western blot for symplekin in Caco-2 cell fractions with or without EGF or U0126 treatment. The protein levels were normalized to those of tubulin and LMNB1, respectively. *P < 0.05, Student’s t-test. ^#^ *P* < 0.05, one-way ANOVA and Student’s t-test. All data are means ± SD, n = 3. Bars, 20 μm.

During wound healing, epithelial cells at the leading edge of the wound gap are migratory, with disrupted cell junctions and polarity, and exhibit certain characteristics of dedifferentiation. Scratch assay on cultured cell monolayer has been used to study the cellular dedifferentiation in various cell lines including highly differentiated cells^18^. To investigate the localization of symplekin in dedifferentiated cells *in vitro*, we performed immunofluorescence (IF) analyses of scratched Caco-2 cell monolayers. Six hours (hr) after wounding, the TJ protein ZO-1 began to translocate to the cytoplasm from cell contacts, and the nuclear localization of symplekin also increased with the impaired junctional staining (Fig. 1A).

To manipulate the differentiation of HT-29 cells *in vitro*, we cultured these cells in glucose- and galactose-containing medium as described previously^19, 20^. Morphological differences could be observed between HT-29/gal and HT-29/glu after 5 days of cultivation, which revealed that colonies of HT-29/gal cells were more compact than HT-29/glu cells (Fig. S1). In the present study, HT-29/gal and HT-29/glu cells were used to model differentiated and dedifferentiated cells, respectively. As shown by IF staining, ZO-1 was distributed along the ridgelines of TJs and symplekin exhibited distinct dual-localization in HT-29/gal cells, whereas in HT-29/glu cells, ZO-1 was diffuse throughout the cytoplasm, and symplekin was restricted to the nucleus (Fig. 1B). Western blotting (WB) analysis also corroborated the above observations, revealing a striking increase in symplekin in the nuclei of HT-29/glu cells, although the total amount of symplekin did not change compared with HT-29/gal cells (Fig. 1C,D).

Caco-2 cells are a highly differentiated cell line. Previous work confirmed that EGF (200 ng/ml) can induce the dedifferentiation of Caco-2 cells^21^. Thus, we detected whether symplekin could be affected in this manner. WB analysis showed that symplekin expression began to increase after 12 hr of EGF stimulation in serum-starved Caco-2 cells (Fig. 1E). To precisely analyse the intracellular redistribution of symplekin, the effect of EGF treatment on Caco-2 cells was monitored at 6-hr time intervals in this study. Caco-2 cells are highly polarized and form typical tight junctions under normal cultivation conditions, and symplekin and ZO-1 showed exclusive TJ IF staining in Caco-2 cells. However, symplekin and ZO-1 partially translocated to the nucleus following EGF stimulation (Fig. 1F, top and middle panels). Moreover, the mitogen-activated protein-kinase kinase (MAPKK, MEK)/extracellular signal-regulated kinase (ERK) pathway is a key downstream mediator of EGF signaling, and inhibition of MEK/ERK with U0126 effectively abrogated the above protein translocation (Fig. 1F, bottom panel). This result was also supported by western blotting of extra-nuclear and nuclear symplekin extracted from Caco-2 cells (Fig. 1G). These findings indicate that the symplekin shuttling from TJs to the nucleus was derived from a pre-existing pool and was not newly synthesized.

### Accumulation of nuclear symplekin promotes cell dedifferentiation and proliferation as well as reduced epithelial tightness

**Figure 2:**
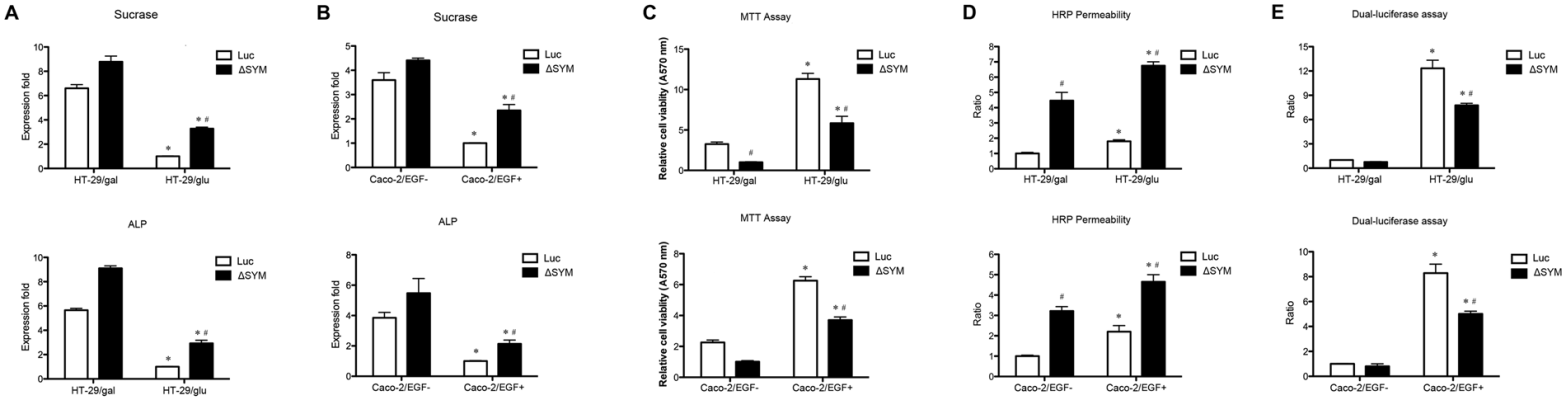
Cellular effects induced by the nuclear accumulation of symplekin. (**A**,**B**) qRT-PCR validation for sucrase and ALP in HT-29 and Caco-2 cells with the indicated treatments. Knockdown of symplekin (ΔSYM) and control (Luc) were also included. MTT assay (**C**) and dual-luciferase assay for Topflash activity (**E**) in HT-29 and Caco-2 cells with the indicated treatments. (**D**) Cell monolayer integrity assessment. The lower chamber medium was collected after 30 min/12 hr permeation for treated HT-29/Caco-2 cells, respectively. Data was analyzed using one-way ANOVA and Student’s t-test. *P < 0.05 versus corresponding differentiation cultured cells. ^#^ *P* < 0.05 versus corresponding RNAi control cells. Values are means ± SEM (n = 3).

Because membrane symplekin is required to support TJs^6^, TJs could not be taken as markers of differentiated epithelial cells in the study. Thus, the brush-border membrane enzymes sucrase and alkaline phosphatase (ALP) were used as differentiated markers to determine enterocytic differentiation^22, 23^. As assayed by quantitative real-time polymerase chain reaction (qRT-PCR), both sucrase and ALP were significantly depressed in HT-29/glu cells (dedifferentiated cells) compared with HT-29/gal cells (differentiated cells). However, the expression of these markers was increased by symplekin reduction, especially in HT-29/glu cells, in which nuclear symplekin was primarily depleted (Fig. 2A). Meanwhile, a similar trend was also observed in EGF-treated Caco-2 cells (Fig. 2B).

In cell proliferation assays, we detected a striking increase in the cell proliferation rate of HT-29/glu cells compared with HT-29/gal cells as well as in EGF-activated Caco-2 cells compared with control cells, which was consistent with previous reports^24^. Furthermore, these effects were largely abrogated by symplekin depletion (Fig. 2C). Taken together, nuclear symplekin appears to play an important role in modulating intestinal cell differentiation and proliferation.

TJs are crucial determinants of epithelial permeability and cell polarity^25, 26^. To study TJ integrity, we performed Boyden chamber permeability assays using horseradish peroxidase (HRP) as a tracer, with the HRP concentration in the medium collected from the lower chamber reflecting the permeability of the epithelial cell monolayer. In accordance with IF staining, HT-29/glu cell monolayers were more permeable than HT-29/gal monolayers after 30 min of cultivation. Similarly, the permeability of Caco-2 cells was enhanced after 12 hr of EGF activation (Fig. 2D).

In addition to growth repression in the dedifferentiated cells, symplekin depletion also reduced the tightness of differentiated cells (HT-29/gal cells and Caco-2 cells), as expected based on its participation in the assembly of TJs in cytoplasmic plaques^6^. Thus, symplekin depletion can induce multiple alterations in cellular functions in various cells.

Another typical shuttle protein, β-catenin, is inclined to traffic to the nucleus and exert oncogenic roles in response to specific cellular signals such as the EGF pathway^27^. In the present study, dual-luciferase assays were performed using the Topflash reporter, and the results showed that the transcriptional activity of β-catenin was strongly increased in HT-29 and Caco-2 cells after dedifferentiation treatment. This effect was impaired after symplekin depletion in both cell types, suggesting the coordination of symplekin and β-catenin function in the nucleus (Fig. 2E).

### Nuclear symplekin regulates cell cycle-related gene expression

**Figure 3:**
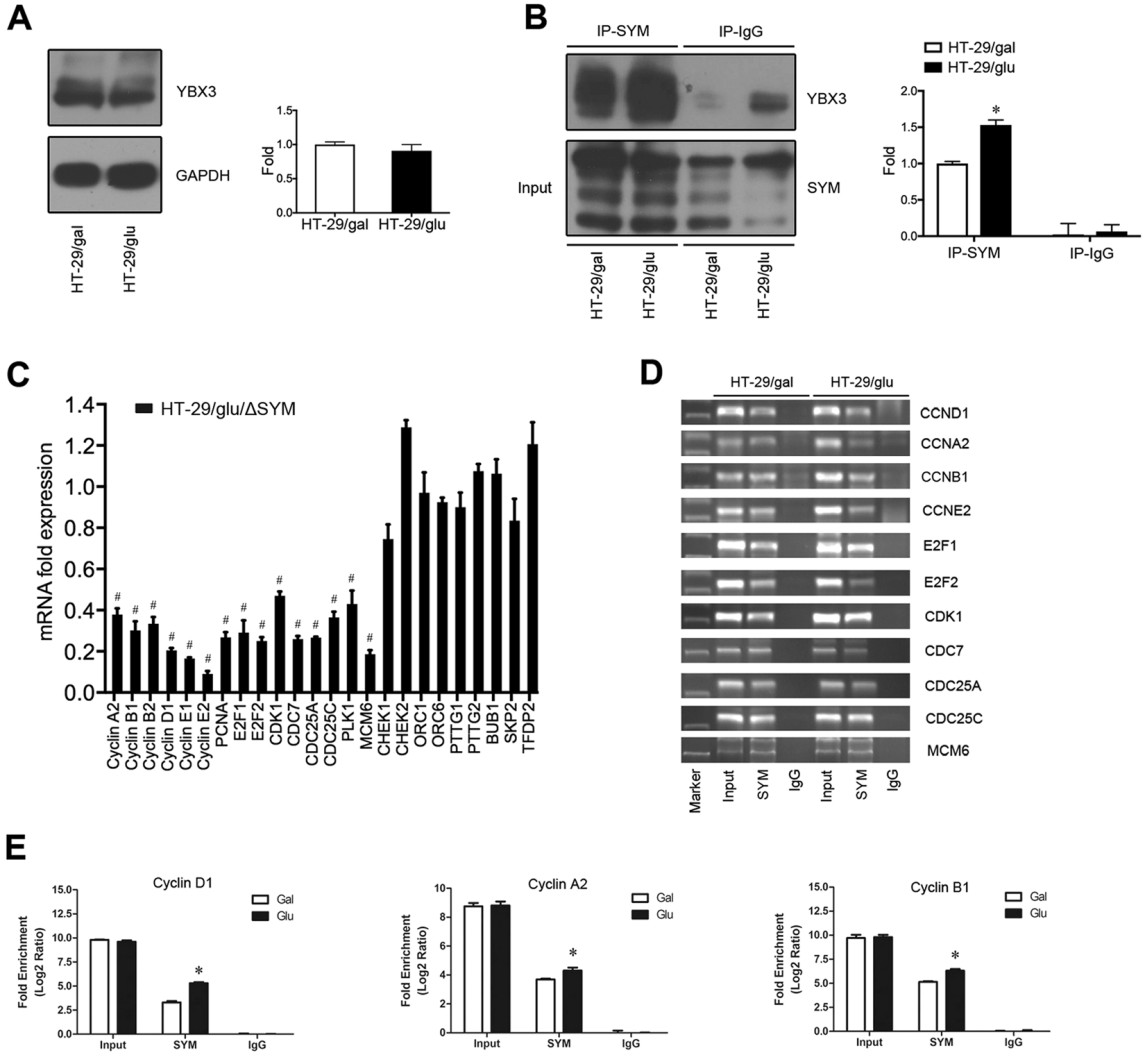
Transcriptional regulation of cell cycle-related genes by nuclear symplekin coupled with YBX3. (**A**) Representative WB bands and relative densitometric quantification of YBX3 expression in HT-29/gal and HT-29/glu cells. (**B**) Endogenous symplekin was immunoprecipitated to identify its binding capacity to YBX3. IgG was used as a negative control. The protein levels were normalized to those of inputs for quantitative analysis. (**C**) qRT-PCR verification of cell cycle-related genes in HT-29/glu/luc and HT-29/glu/ΔSYM cells. mRNA expression in HT-29/glu/ΔSYM cells was normalized to those in HT-29/glu/luc cells. (**D**) PCR amplification of DNA fragments precipitated by a symplekin antibody and IgG in HT-29/gal and HT-29/glu cells; total sonicated chromatin prior to ChIP was taken as a control. (**E**) q-PCR detection of cyclin D1, cyclin A2, and cyclin B1 promoter enrichment in ChIP DNA products prepared from HT-29/gal and HT-29/glu cells using the indicated antibodies. *P < 0.05 in comparison to the value in HT-29/gal cell with anti-symplekin precipitation, one-way ANOVA and Student’s t-test. ^#^ *P* < 0.05 in comparison to the indicated gene expression in control cells, Student’s t-test. Results are means ± SD, n = 3.

Symplekin has been reported to bind the transcription factor YBX3 and to play roles in cell proliferation^2^. The nuclear localization of YBX3 is prominent in low-density proliferating cells^16^. In the present study, we first affirmed that expression of YBX3 was stable in HT-29 cells cultured under different conditions (Fig. 3A). To test whether interactions between symplekin and YBX3 were correlated with different cell states, we performed co-immunoprecipitation (Co-IP) experiments using isolated nuclear proteins, and the results showed that the endogenous binding of symplekin to YBX3 was increased in HT-29/glu cells compared with HT-29/gal cells (Fig. 3B). The binding of symplekin to YBX3 may active the transcription of downstream genes.

To investigate the transcriptional regulatory roles of nuclear symplekin, we performed mRNA microarrays to identify differentially expressed genes between HT-29/glu/ΔSYM cells (symplekin RNAi in dedifferentiated cells, in which mainly nuclear symplekin was depleted) and HT-29/glu/luc (RNAi control, in which symplekin is primarily localized to the nucleus). Most of the differentially expressed genes were enriched among cellcycle-related processes according to the Gene Ontology (GO) and the Kyoto Encyclopedia of Genes and Genomes (KEGG) databases (Figs S2 and S3). qRT-PCR analysis confirmed that the expression of 15 genes (cyclin A2, cyclin B1, cyclin B2, cyclin D1, cyclin E1, cyclin E2, PCNA, E2F1, E2F2, CDK1, CDC7, CDC25A, CDC25C, PLK1 and MCM6) was significantly modified among the clustered genes (Fig. 3C).

YBX3 binds the inverted CCAAT box sequence^16^. In addition to known regulated genes such as ErbB2, cyclin D1 and PCNA, we identified inverted CCAAT boxes in the promoters of all of the above differentially expressed genes except for cyclin B2 and PLK1. We then performed chromatin immunoprecipitation (ChIP) assays using a symplekin antibody and detected symplekin/YBX3 complexes bound to the promoters of 11 genes (cyclin A2, cyclin B1, cyclin D1, cyclin E2, E2F1, pE2F2, CDK1, CDC7, CDC25A, CDC25C and MCM6) in HT-29/gal and HT-29/glu cells (Fig. 3D). Furthermore, quantitative PCR (q-PCR) analyses conducted using oligonucleotides derived from the promoters of three genes (cyclin A2, cyclin B1 and cyclin D1) that were precipitated by a symplekin antibody demonstrated that DNA enrichment was increased in HT-29/glu cells compared with HT-29/gal cells (Fig. 3E).

### Phosphorylated symplekin is enriched in the nucleus

**Figure 4:**
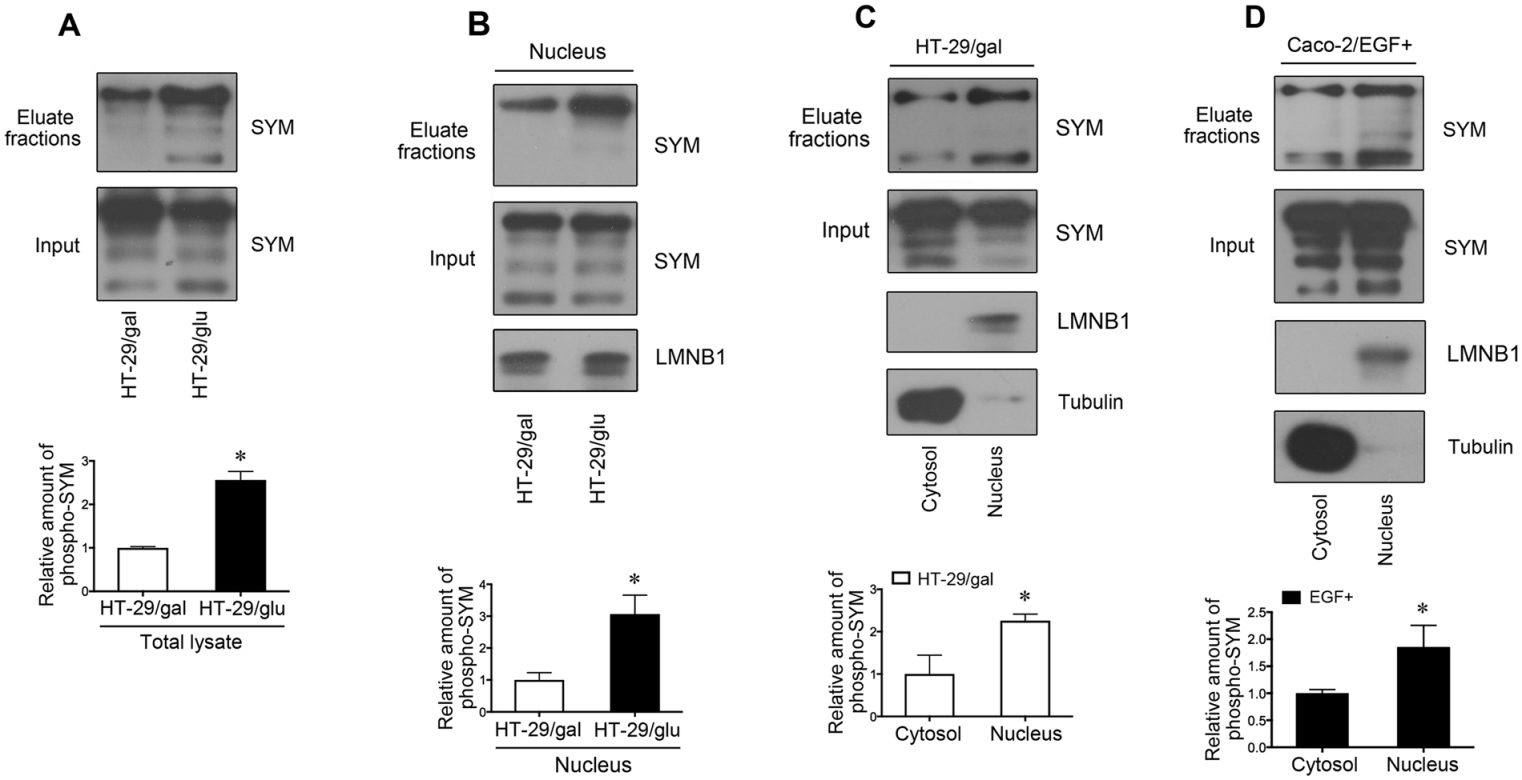
Determination of phosphorylated symplekin in cells. (**A**) Cell protein extraction, phosphoprotein purification, and WB assay for symplekin (SYM) in HT-29/gal and HT-29/glu cells. (**B**) WB bands of symplekin in nuclear phosphoproteins isolated from HT-29/gal and HT-29/glu cells. (**C**) Comparison of phosphorylated symplekin between HT-29/gal cytosol and nucleus. (**D**) Comparison of phosphorylated symplekin between EGF stimulated Caco-2 cell cytosol and nucleus. Tubulin and LMNB1 were employed to determine the cell fractionation efficiency. Phosphoproteins were isolated from the eluate fraction. Total cell or nuclear proteins before phospho-purification were used as the input. Phosphorylated symplekin was normalized to the levels of input symplekin in total cellular, cytosolic, or nuclear lysates, and graphic representation of relative amount of phosphorylated symplekin (phospho-SYM) was shown in the bottom panels. ^#^ *P* < 0.05, Student’s t-test. Data are means ± SD, n = 3.

Cytoplasmic proteins can be phosphorylated by activated kinases and then translocate into the nucleus to regulate cellular processes^28^. To understand whether the phosphorylation of symplekin is related to its distribution between epithelial TJs and the nucleus, we inspected and compared the levels of phosphorylated symplekin between dedifferentiated and differentiated cells as well as between nuclear and cytoplasmic fractions. Symplekin phosphorylation was significantly increased in HT-29/glu cells compared with HT-29/gal cells, as determined by western blotting analyses of phosphoproteins purified by a symplekin antibody (Fig. 4A). Meanwhile, the comparison between their nuclear lysates also showed a higher phosphorylation amount of nuclear symplekin in HT-29/glu than that in HT-29/gal cells (Fig. 4B). Furthermore, analysis of the nuclear and cytoplasmic/membrane fractions in polarized HT-29/gal cells indicated that phosphorylated symplekin was primarily localized to the nucleus (Fig. 4C); a similar result was also obtained using EGF-treated Caco-2 cells (Fig. 4D). Based on these observations, we concluded that the phosphorylation of symplekin might be associated with its subcellular distribution.

### The nuclear translocation of symplekin is dependent on ERK phosphorylation

MAPK/ERK regulates a number of cellular processes by phosphorylating serine and threonine residues in target proteins^29^. Bio-informatic prediction using Scansite (http://scansite3.mit.edu) and sequence analyses identified several putative residues corresponding to ERK D-domains and ERK-kinase motifs within the amino acid sequence of symplekin. Because ERK substrates usually have docking domains and substrate motifs^30^, and in view of the fact that nuclear symplekin showed a higher degree of phosphorylation, we speculated that ERK1/2 might regulate symplekin nuclear translocation. Therefore, we investigated the relationship between ERK1/2 phosphorylation and symplekin nuclear translocation.

**Figure 5:**
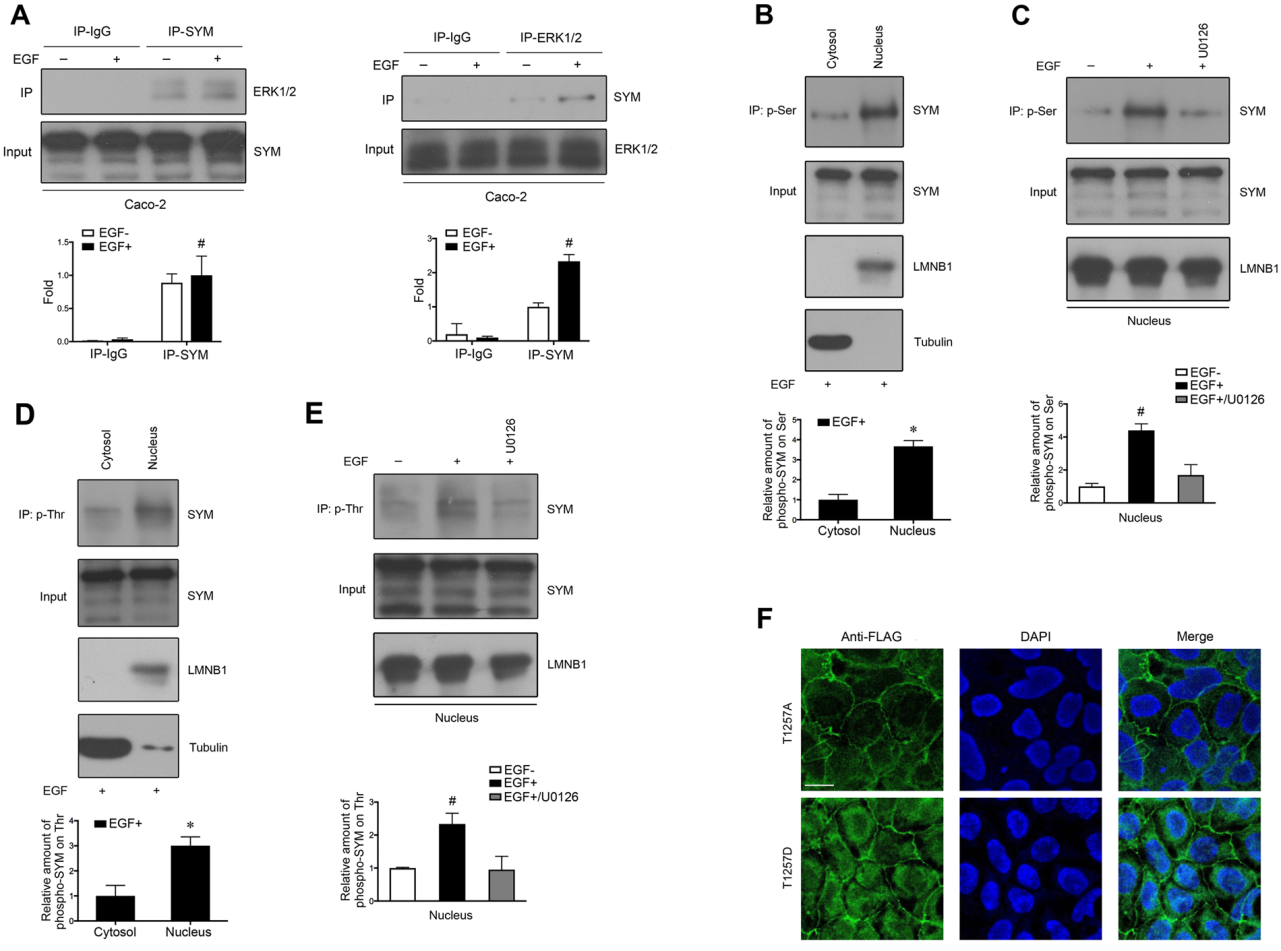
Phosphorylation and subcellular localization of symplekin regulated by ERK. (**A**) Forward and reciprocal Co-IP between symplekin (SYM) and ERK1/2. WB was carried out with antibodies against symplekin or ERK1/2 in Caco-2 cells with or without EGF treatment. Total cell lysates (input) were used as the loading control for quantitative analysis. (**B**,**D**) IP using anti-phosphoserine (p-Ser) and phosphothreonine (p-Thr) antibodies in cytoplasmic/membrane (Cytosol) and nuclear (Nucleus) lysates prepared from Caco-2 cells with EGF treatment. The IP input was taken as the normalized control for phosphorylated symplekin quantification. Tubulin and LMNB1 were used as markers for cell fractions. (**C**,**E**) IP using anti-phosphoserine and phosphothreonine antibodies in nuclear lysates (Nucleus) from Caco-2 cells receiving the indicated treatments. U0126 (40 μM) was added for 30 min before EGF stimulation. The IP input was taken as the loading control for quantitative analysis. LMNB1 was shown as a nuclear marker. ^*^ *P* < 0.05, Student’s t-test. ^#^ *P* < 0.05, one-way ANOVA and Student’s t-test. All data are means ± SD, n = 3. (**F**) IF analyses were performed with an anti-Flag antibody to detect the subcellular localization of symplekin mutants. Nuclei were stained with DAPI. Bars, 20 μm.

Based on the above result that EGF treatment leads to symplekin accumulation in the nucleus of Caco-2 cells (Fig. 1F), we used EGF-treated Caco-2 cells to study the mechanisms of symplekin translocation and obtained the following results. 1) Co-IP assays showed that symplekin and ERK1/2 were able to bind each other, and this binding was enhanced by EGF treatment (Fig. 5A, left). Reciprocal Co-IP analysis also yielded the same results (Fig. 5A, right). 2) EGF stimulation resulted in increased serine and threonine phosphorylation of nuclear symplekin, which was abrogated by the MEK/ERK inhibitor U0126 (Fig. 5B–E), indicating that ERK1/2-regulated symplekin phosphorylation can be induced by EGF treatment. 3) The nuclear translocation of symplekin was effectively abrogated by the above U0126 treatment (Fig. 1F). 4) We utilized mass spectrometry to define the phosphorylation sites on symplekin. In symplekin immunoprecipitated from nuclear extracts and total cellular proteins, T1257 was identified as a phosphorylation site in EGF-treated Caco-2 cells, which was consistent with the predicted ERK-kinase motif. Flag-tagged phosphodeficient T1257A and phosphomimetic T1257D were then transfected into Caco-2 cells to test their function. According to IF staining, T1257A localized to TJs and the cytoplasm, whereas T1257D showed increased nuclear accumulation, although its TJ localization was detectable as well (Fig. 5F). However, no obvious differences were observed among wild-type symplekin and the mutants regarding their ability to bind YBX3 in the same cell line (Fig. S5). Taken together, these results suggested that ERK1/2 might play a role in the nuclear translocation of symplekin via the phosphorylation of specific residues.

### Symplekin depletion impairs cell junctional complexes

**Figure 6:**
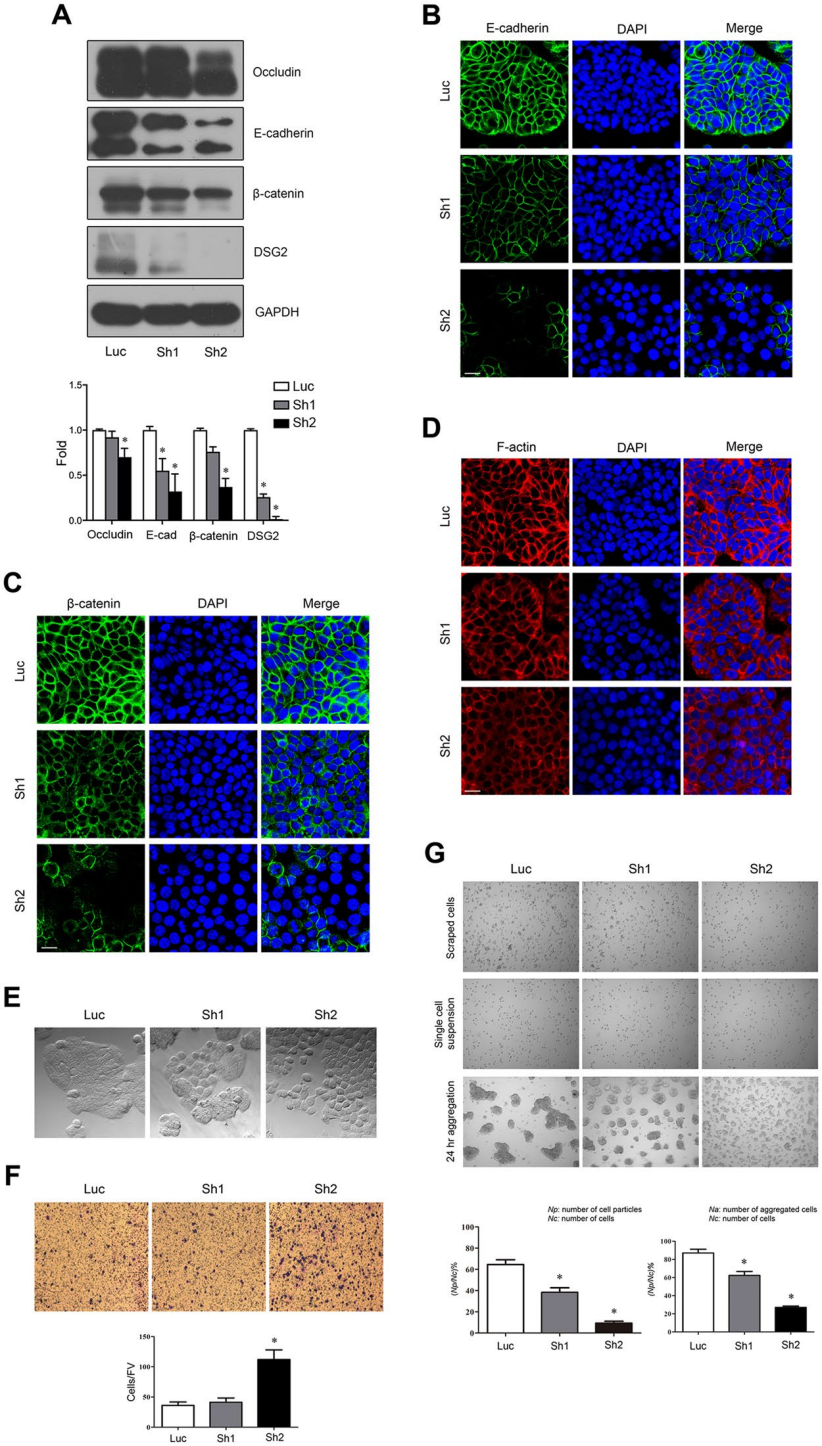
Epithelial junctional complexes affected by symplekin depletion. (**A**) WB bands and quantification analysis for expressions of occludin, E-cadherin, β-catenin, and DSG2 in stable symplekin-silenced HT-29 cells (Sh1 and Sh2) and the control (Luc). (**B**,**C**) IF staining of E-cadherin and β-catenin in the above stable clones. DAPI showed the nuclei staining. (**D**) Cytoskeletal F-actin staining using rhodamine phalloidin. (**E**) Morphological comparison among indicated HT-29 cell colonies captured by a phase-contrast microscopy. (**F**) Matrigel invasion assays in HT-29 cells with or without symplekin knockdown. The migrated cells were stained with crystal violet and counted in six random fields. FV, field vision. (**G**) Cell disassociation and aggregation analyses. Six random fields were chosen for each group. *P < 0.05, one-way ANOVA and Student’s t-test. All results are means ± SD, n = 3. Bars, 20 μm.

Roles for symplekin in preserving TJs and cell polarity have been revealed previously^6^. Here, the expressions of a TJ molecule (occludin), adherens junction proteins (E-cadherin and β-catenin), and the desmosome protein desmoglein 2 (DSG2) were decreased in stable symplekin-depleted HT-29 cells (stable clones of Sh1, Sh2, and the control Luc, and their corresponding symplekin levels were described previously)^6^ compared with the control according to WB and IF staining (Fig. 6A,B). Additionally, β-catenin and the cortical F-actin cytoskeleton were partially diffused in the cytoplasm after symplekin depletion (Fig. 6C,D).

Cultured HT-29 cells were polarized and were observed to form compact colonies under phase contrast microscopy; however, symplekin depletion resulted in extended bulk and loose contacts between cells, which was consistent with the previously characterized “metastable phenotypes”^31^ (Fig. 6E). Furthermore, transwell assays showed that the “metastable cells” displayed increased migration through Matrigel pads compared with the control (Fig. 6F). Cell disassociation and aggregation analyses confirmed that symplekin depletion significantly dampened cell adhesion (Fig. 6G). Collectively, these results indicate that TJ-associated symplekin is required to maintain the integrity and function of epithelial junctional complexes.

## Discussion

The human colon adenocarcinoma cell lines HT-29 and Caco-2 are able to undergo spontaneous enterocytic differentiation and form microvilli under certain culture conditions after reaching confluence and therefore represent useful models to study cell junctions and cell polarity *in vitro* ^32, 33^. Although HT-29 cells exhibit highly differentiated characteristics in galactose-containing medium, some dedifferentiated properties are retained in glucose-containing medium^19, 20^; meanwhile, Caco-2 cells exhibit poorly differentiated features after treatment with certain concentrations of EGF^21^. In the present study, we found that TJ-associated symplekin translocated to the cellular nucleus in dedifferentiated cells along with an increased proliferation rate but without remarkable alternations in its total expression level *in vitro*, which is consistent with previous results obtained from normal human colonic epithelium and colorectal tumor samples *in vivo* ^3, 5^.

Furthermore, the functions and mechanisms of nuclear symplekin accumulation were investigated. YBX3 is another TJ-associated and dual-localized protein with known roles in regulating cell proliferation through transcriptional modulation and interactions with CDK4^34^. The functional interaction of YBX3 with symplekin has also been confirmed^2, 16^. In our study, the binding of nuclear symplekin to YBX3 was increased in dedifferentiated HT-29 cells (HT-29/glu), along with significantly up-regulated proliferation. Because nuclear symplekin plays a dominant role in HT-29/glu cells, exogenous expression of this protein is expected to primarily mirror the relevant downstream alternations caused by nuclear symplekin. As expected, our assays confirmed that nuclear symplekin exerts pervasive regulatory roles, especially in cell growth and proliferation. Aside from cyclin D1 and PCNA^7^, several genes (cyclin A2, cyclin B1, cyclin D1, cyclin E2, E2F1, pE2F2, CDK1, CDC7, CDC25A, CDC25C and MCM6) involved in all four phases of the cell cycle were identified as being regulated by the symplekin/YBX3 transcriptional complex. TJ-associated and dual-localization proteins have previously been shown to modulate cell proliferation by promoting the G1/S phase transition^7^, and our observations provide a parallel mechanism for the modulation of cell cycle progression by nuclear symplekin, which serves here as a transcriptional co-factor in addition to its well-characterized function in mRNA maturation.

Cell proliferation and differentiation are closely linked physiological processes. In general, poorly differentiated cells are inclined to exhibit high rates of proliferation. The present study also investigated the roles of nuclear symplekin in enterocyte differentiation. Based on previous models^20, 21^, we further confirmed that the nuclear accumulation of symplekin reduce the expression levels of epithelial cell differentiation markers. The activation of specific genes is known to regulate cell proliferation and dedifferentiation simultaneously, such as cyclin D1^35, 36^, one of the downstream genes of the symplekin/YBX3 transcriptional complex. Taken together, we hypothesize that nuclear symplekin acts as a transcriptional co-factor in cooperation with YBX3 to promote cell proliferation and dedifferentiation by regulating gene expression.

Apart from binding to YBX3, the association between symplekin and ZO-1 was demonstrated previously^6^, suggesting that three TJ-associated proteins, YBX3, symplekin, and ZO-1, may interact to regulate the cell cycle- and epithelial differentiation-related processes^2, 16^. For example, ErbB-2 is a tyrosine kinase co-receptors that plays multiple roles in cell proliferation and differentiation and whose expression is frequently increased in various tumors^37, 38^. In the present study, we found that enriched ErbB-2 promoter fragments appeared in DNA products precipitated using a symplekin antibody (data not shown). In view of these findings and the fact that ErbB-2 transcription can be modulated by interacting ZO-1 and YBX3^16^, we speculate that ErbB-2 may also be a crucial downstream effector of symplekin to affect cell proliferation and differentiation.

**Figure 7:**
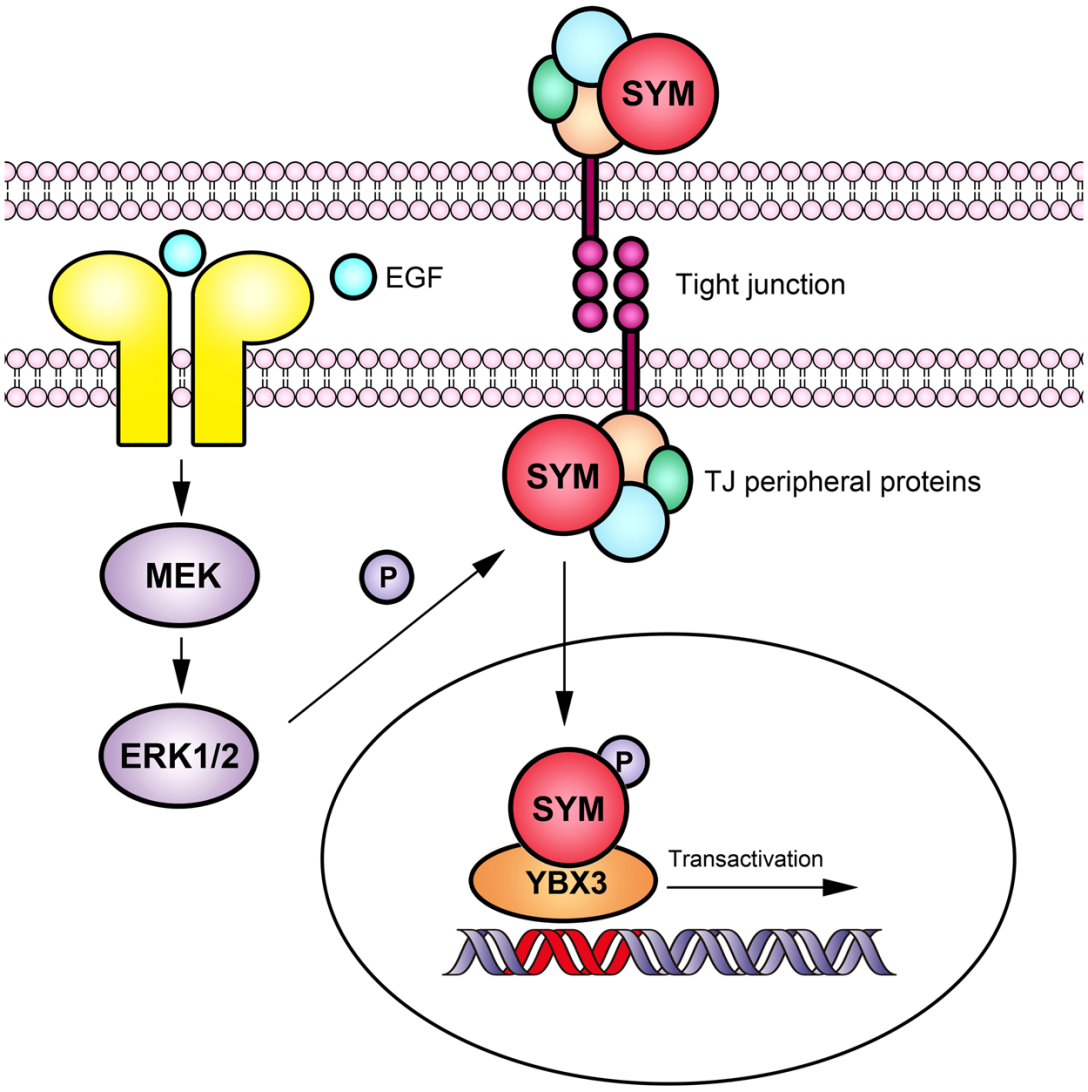
Schematic representation of the ERK-dependent regulation of symplekin function.

The nuclear translocation of symplekin showed functional significance, as described above. Thus, we studied its regulatory mechanisms. First, we compared status of nuclear and extra-nuclear symplekin and found that nuclear symplekin displayed increased phosphorylation compared with the tight junction pool. Phosphorylation is among the most fundamental and well-studied post-translational modifications in modulating protein functions and takes part in a wide range of cellular processes, especially in signal transduction^39^. Protein phosphorylation has also been described in regulating the dynamic properties of tight junction components. ZO-1 phosphorylation by protein kinase C (PKC) required for the formation of cell contacts^40^. Threonine phosphorylation on occludin has been confirmed to induce its disassociation from tight junctions^41^. Increases and decreases in tight junction assembly are also linked with diverse phosphorylation on claudins^42^. Subsequently, we investigated the significance and mechanisms of symplekin phosphorylation. Our data demonstrated that the nuclear translocation of symplekin was regulated by means of, but might not be limited to, an ERK1/2 dependent mechanism through the phosphorylation of symplekin at Thr-1257, after which nuclear symplekin conducts its nuclear functions through increased binding to YBX3 (Fig. 7).

As a peripheral component of tight junctions, the shuttling of YBX3 between the cytoplasm and nucleus still remains poorly understood, while our current observations highlight symplekin as an important multifunctional mediator to transduce extracellular signals into the nucleus in a manner similar to that of β-catenin during Wnt signaling^12^. Given that β-catenin is a downstream effector of EGFR^27^ and that symplekin reduction impaired β-catenin activation in the nucleus in the present study and along with the finding that these proteins have certain overlapping target genes such as cyclin D1^43^, we speculate that symplekin and β-catenin might play coordinated or compensatory roles. In addition, nuclear β-catenin is a negative modulator of ZO-1 expression^44^, and ZO-1 down-regulation might also facilitate the dissociation of symplekin from TJs to enter the nucleus. Thus, nuclear symplekin and β-catenin might cooperate to form a positive feedback loop supporting dedifferentiation and proliferation in dedifferentiated epithelial cells.

Epithelial intracellular junctions, including TJs, adherens junctions and desmosomes, typically show structural and functional correlation^15^. In the process of epithelial-mesenchymal transition (EMT), TJs disassociate during the first stage, followed by the disassembly of other basolateral junctions, and their assembly order is reversed in the course of the mesenchymal-epithelial transition (MET)^45^. Here, we found that, in addition to assembling TJs^6^, symplekin is also obligatory for the stability of adherens junctions. The expression and localization of adherens molecules were significantly affected by symplekin depletion in cultured cells, which might have resulted from the disruption of apically located junctions or transcriptional alternations, although epithelial traits were still partially retained. However, more specific mechanisms still require further elucidation.

Considering that symplekin expression differs in various types of cancer^5, 46^, symplekin might play dual roles during tumor progression. At the cell membrane, symplekin serves as a peripheral ingredient to support cell junctions and polarity; in the nucleus, symplekin modulates cell proliferation and dedifferentiation via interactions with YBX3 mediated by the phosphorylation of specific residues. The decreased presence of TJ symplekin and the increased accumulation of nuclear symplekin play dual roles to reduce cell adherence and polarity and induce cell proliferation and dedifferentiation, which may participate in tumorigenesis. Understanding the roles and regulatory mechanism of symplekin is of significance to comprehend tumor initiation and development and to seek effective diagnostic and therapeutic approaches to treat various cancers.

## Methods

### Antibodies

Antibodies were purchased from the following companies: two symplekin monoclonal antibodies (mAbs) were from Progen Biotechnik (cat #651100; Heidelberg, Germany) and BD Biosciences (cat #610644; Rockville, MD, USA); ZO-1 mAb (HPA001637), CSDA mAb (SAB1404593), GAPDH mAb (G8795), rabbit anti-FLAG (F7425) antibody, FITC-conjugated goat anti-rabbit Immunoglobulin G (IgG) antibody (F6005), anti-flag affinity gel (F2426), and 3 × FLAG Peptide (F4799) were from Sigma-Aldrich (St. Louis, MO, USA); ERK1/2 mAb (13–6200) and rabbit anti-β-catenin (AHO0462) antibody were from Invitrogen (Carlsbad, CA, USA); biotinylated rabbit anti-phosphoserine (ab9335) and rabbit anti-phosphothreonine (ab9340) antibodies were from Abcam (Cambridge, MA, USA); rabbit anti-E-cadherin (3195S) antibody was from Cell Signaling Technology (Beverly, MA, USA); tubulin-α mAb (66031) and LMNB1 mAb (66095) were from Proteintech (Wuhan, China); Cy3-conjugated goat anti-mouse IgG antibody (115-165-166) was from Jackson ImmunoResearch Laboratories (West Grove, PA, USA); peroxidase-conjugated goat anti-mouse IgG antibody (HD003-1) was from DingGuo (Beijing, China); and peroxidase-conjugated goat anti-rabbit IgG antibody (M21002) was from Abmart (Shanghai, China).

### Cell culture

The human epithelial colorectal adenocarcinoma cell lines HT-29 and Caco-2 were acquired from the Kunming Cell Bank of Type Culture Collection, Chinese Academy of Sciences (Kunming, Yunan, China). HT-29 and Caco-2 cells were cultured in RPMI 1604 medium (Gibco, Grand Island, NY, USA) and Dulbecco’s modified Eagle’s medium (DMEM) (Hyclone, Logan, UT, USA), respectively. Both media contained 10% fetal bovine serum (FBS), 1 mM pyruvate and 1 × NEAA (cat #11140050, Gibco).

Differentiated HT-29 cells were cultured as described previously, with minor modifications^20^. Briefly, glucose and pyruvate-free RPMI 1640 culture medium (cat #11879-020, Gibco) with 10% FBS was repeatedly supplemented with 20 mM glucose or 5 mM galactose for HT-29 culture. The numbers of dead cells did not significantly increase after transfer from normal RPMI 1640 medium to the indicated glucose or galactose medium. Cell-based assays were carried out after at least 5 days of cultivation. For EGF treatment, Caco-2 cells were pre-starved in serum-free medium for 10 hr and then stimulated with EGF (200 ng/ml; Millipore, Bedford, MA, USA).

### qRT-PCR

Total RNA extraction, cDNA preparation and qRT-PCR were performed as reported previously^47^. The corresponding primer sets are described in Table S1.

### IF staining

Caco-2 cells were grown on cover slides treated with 0.01% poly-L-lysine (Sigma-Aldrich); HT-29 cells were grown on slides coated with 0.5-mm thick Matrigel (BD Biosciences). After fixation with cold acetone, the cell slides were blocked and incubated with the indicated antibodies, followed by 4′,6-diamidino-2-phenylindol (DAPI) staining. F-actin was stained with rhodamine-conjugated phalloidin (Invitrogen). The mounted slides were then visualized and captured on a confocal laser scanning microscope.

### WB and IP

Total cellular proteins were extracted using RIPA buffer (Beyotime, Shanghai, China) containing protease inhibitors. The lysates were resolved via 8–12% sodium dodecyl sulfate polyacrylamide gel electrophoresis (SDS-PAGE) and transferred onto PVDF membranes (Millipore). The membranes were then blocked and incubated with the indicated antibodies for immuno-blotting. The blot scans on X-ray films were processed in ImageJ software (NIH, Bethesda, MD, USA) for quantitative measurements.

For Co-IP, cells were lysed in IP buffer (20 mM HEPES, pH 8.0, 1.5 mM MgCl_2_, 0.2 mM EDTA, 0.5% NP40, and 10% glycerol) supplemented with 600 mM NaCl and were then diluted with 3-fold with IP buffer after treatment with benzonase nuclease (Sigma-Aldrich). Aggregated proteins were removed by centrifugation, and the lysates were then incubated with the indicated antibodies and protein beads.

For phosphoserine/phosphothreonine IP, pelleted cell nuclei were lysed in buffer I (50 mM Tris-HCl, pH 7.5, 150 mM NaCl, and 1.5% SDS). After sonication, the lysates were boiled, centrifuged and diluted 10-fold with buffer II (50 mM Tris-HCl, pH 7.5, 150 mM NaCl, 0.5% NP-40, and 0.5% bovine serum albumin (BSA) with protease and phosphatase inhibitors). The lysates were incubated with biotin-conjugated anti-phospho-Ser/Thr antibodies, and the immuno-complexes were isolated using streptavidin beads (Dynabeads M-280; Invitrogen). Cell cytoplasmic lysates were boiled with 0.3% SDS and processed for IP.

### mRNA expression profiling

mRNA profiling was performed in HT-29 cells using the GeneChip PrimeView Human Gene Expression Array (Affymetrix, Santa Clara, CA, USA) by CapitalBio Corporation (Beijing, China; http://www.capitalbio.com). The enriched differentially expressed genes were analyzed by GO and KEGG pathway analyses using the Mas 3.0 molecule annotation system (http://bioinfo.capitalbio.com/mas3).

### RNA interference

Symplekin RNA interference was achieved by cell infection using designed retrovirus (targeting TCTGGTCCTCATCAGCATG). The virus collection, cell infection and HT-29 stable clone screening (luc, sh1 and sh2) were described in our previous study^6^.

### Dual-luciferase assay

A total of 2.5 × 10^4^ cells were seeded in sextuplicate into a 96-well plate, and after 24 hr, 90 ng of SuperTOPflash and 10 ng of pRL-TK plasmid well were transfected into each. Forty-eight hr later, cells were lysed, and luciferase activity was measured using a Dual-Luciferase assay kit (Promega). TOPflash luciferase activity was normalized to that of Renilla luciferase. For Caco-2 cells, 200 ng/ml EGF was added for the indicated times before harvesting.

### Subcellular fractionation

Cell nuclear and cytosolic fractions were isolated using the nuclear extract kit (Active Motif, Carlsbad, CA, USA). The subcellular fractions were subjected to Co-IP, phosphoprotein isolation, and western blotting analysis. Protein concentrations were determined using a BCA protein assay kit (Beyotime).

### Phosphoprotein isolation

Cell phosphoprotein isolation was carried out using a phosphoprotein purification kit (Qiagen, Hilden, Germany). Briefly, cells or nuclear fractions were washed with HEPES buffer and scraped off for lysis in CHAPS buffer, after which 2.5 mg of total protein lysate was subjected to purification on the columns, and phosphoproteins were then eluted and quantified.

### Mass spectrometry analysis

Endogenous symplekin was purified by IP. Proteins in the gel band were excised and in-gel digested with trypsin. Eluted peptides were subjected to liquid chromatography performed on a nano Acquity UPLC system (Waters Corporation, Milford, MA, USA) connected to a Triple TOF™ 4600 mass spectrometer (AB SCIEX, Framingham, MA, USA). The mass spectra were searched using Mascot Daemon software (Version 2.3.02, Matrix Science, London, UK) based on the UniProtKB/Swiss-Prot database.

### DNA construct mutagenesis and cell transfection

The symplekin-pcDNA 3.1-FLAG mutant constructs were created via a site-directed mutation with PCR-driven overlap extension^48^. Cell transfection was carried out using the FuGENE@ HD transfection reagent (Promega, Madison, WI, USA) according to the manufacturer’s instructions. The ratio of transfection reagent to DNA was 3:1.

### ChIP

ChIP was performed using a symplekin antibody and the EZ-Magna ChIP A/G kit (Millipore) as described previously^49^. PCR amplifications for controls were included to test the efficiency of the experiment. The primer sets used for the precipitated sequences analysis are described in Table S1.

### Cell proliferation assay

Cell proliferation assays were performed based on the 3-(4, 5-dimethylthiazol-2-yl)-2, 5-diphenyltetrazolium bromide (MTT) method. A total of 4 × 10^3^ cells were seeded in triplicate in a 96-well plate. After 24 hr, the medium was replaced with 100 µl of fresh medium, and MTT was added at 10 µl per well. After incubation, the medium was removed, followed by the addition of dimethyl sulfoxide (DMSO). The absorbance was read at 570 nm on a microplate reader.

### Cell monolayer integrity assay

Cell monolayer integrity was measured by the permeability of horseradish peroxidase (HRP) through the Boyden chamber^45^. Briefly, cells were cultured complete confluence on permeable filters (with 0.4-μm pores). The medium in the upper chamber was then replaced with HRP-containing medium, and the basal chamber medium was harvested at different time points. Then, the substrate solution (50 mM sodium phosphate buVer, pH 5.0, 342 M o-dianisidine dihydrochloride, 0.003% H_2_O_2_) was added, followed by the addition of 0.1% sodium azide. Optical density was read at 450 nm.

### Cell disassociation and aggregation assay

Experiments on cell disassociation were performed as described previously, with modifications^50^. Confluent HT-29 cells grown in a well were treated with 0.25% trypsin containing 1 mM CaCl_2_ for 30 min at 37 °C and pipetted gently 10 times with culture medium. The number of cell particles was counted in six random visual fields. Cells grown in a parallel well were treated with trypsin/EDTA to record the total cell number.

For aggregation assays, cells treated with trypsin/CaCl_2_ were pipetted and filtered through a cell strainer (Miltenyi Biotec, Bergisch-Gladbach, Germany). Single-cell suspensions in 10% Matrigel were seeded into a 12-well plate coated with 0.5-mm-thick Matrigel for 24 hr to collect the number of aggregated cells under a microscope from six random fields. Cells in a parallel well without coating were digested to record the total cell number.

### Cell invasion assay

A total of 1 × 10^5^ starved cells suspended in 400 µl of serum-free medium were seeded into the upper chamber of each hanging cell culture insert (8-µm pore size, Millipore) coated with 100 µl diluted Matrigel in a 12-well plate. Twelve hr later, the gel was wiped off, and the filters were fixed and stained with crystal violet. Invaded cells were examined and counted on a light microscope.

### Statistical analysis

All the data are presented as the mean ± standard deviation (SD) of at least three independent experiments. The data were analyzed using SPSS 17.0 software. The measurement data were analyzed using Student’s t-test or one-way/two-way analysis of variance (ANOVA) as appropriate. Statistical significance was set at 0.05 for all tests.

### Data availability

The datasets generated during the current study are available from the corresponding author on reasonable request.

## Electronic supplementary material

Supplementary Information
