# Supplementary material for: Nuclear accumulation of symplekin promotes cellular proliferation and dedifferentiation in an ERK1/2-dependent manner
Source: Sci Rep. 2017 Jun 19;7:3769. doi: 10.1038/s41598-017-04005-z (PMC5476558; doi:10.1038/s41598-017-04005-z)
Supplement: Supplementary file 1 — Supplementary Information [file 41598_2017_4005_MOESM1_ESM.pdf]

Nuclear accumulation of symplekin promotes cellular proliferation and dedifferentiation in an ERK1/2-dependent manner

[1,2] Chen Zhang, [3] Hai-Lei Mao, [1] Yi Cao

[1] Laboratory of Molecular and Experimental Pathology, Kunming Institute of Zoology, Chinese Academy of Sciences, Kunming, China

[2] Kunming College of Life Science, University of Chinese Academy of Sciences, Kunming, China

[3] Department of Anesthesiology and Critical Care Medicine, Zhongshan Hospital, Fudan University, Shanghai, China

Address for correspondence:

Prof. Dr. Yi Cao

Laboratory of Molecular and Experimental Pathology

Kunming Institute of Zoology

Chinese Academy of Sciences

32 Jiaochang Donglu, Kunming

Yunnan 650223

China.

Tel.: 0086-871-65199081

Fax: 0086-871-65199081

E-mail: caoy@mail.kiz.ac.cn

Supplementary Information

Figure S1. Morphological observation of HT-29/gal and HT-29/glu cells under light microscope.

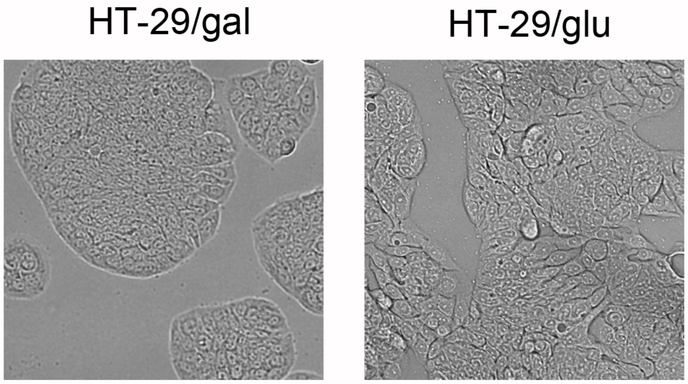

Figure S2. Heat-map of differentially expressed genes enriched during cellular proliferation induced by symplekin silencing. Green, down-regulation; red, up-regulation.

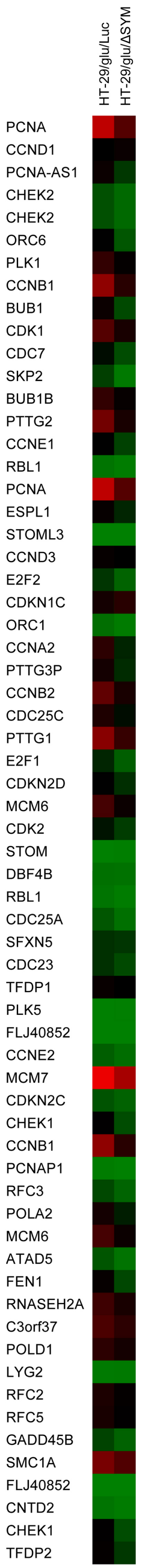
HT-29/glu/Luc
HT-29/glu/ΔSYM
PCNA
CCND1
PCNA-AS1
CHEK2
CHEK2
ORC6
PLK1
CCNB1
BUB1
CDK1
CDC7
SKP2
BUB1B
PTTG2
CCNE1
RBL1
PCNA
ESPL1
STOML3
CCND3
E2F2
CDKN1C
ORC1
CCNA2
PTTG3P
CCNB2
CDC25C
PTTG1
E2F1
CDKN2D
MCM6
CDK2
STOM
DBF4B
RBL1
CDC25A
SFXN5
CDC23
TFDP1
PLK5
FLJ40852
CCNE2
MCM7
CDKN2C
CHEK1
CCNB1
PCNAP1
RFC3
POLA2
MCM6
ATAD5
FEN1
RNASEH2A
C3orf37
POLD1
LYG2
RFC2
RFC5
GADD45B
SMC1A
FLJ40852
CNTD2
CHEK1
TFDP2

Figure S3. Enrichment analysis of KEGG-pathways and GO terms for differentially expressed genes between HT-29/gal and HT-29/glu cells. (*P* < 0.05, two-sided Fisher's exact test).

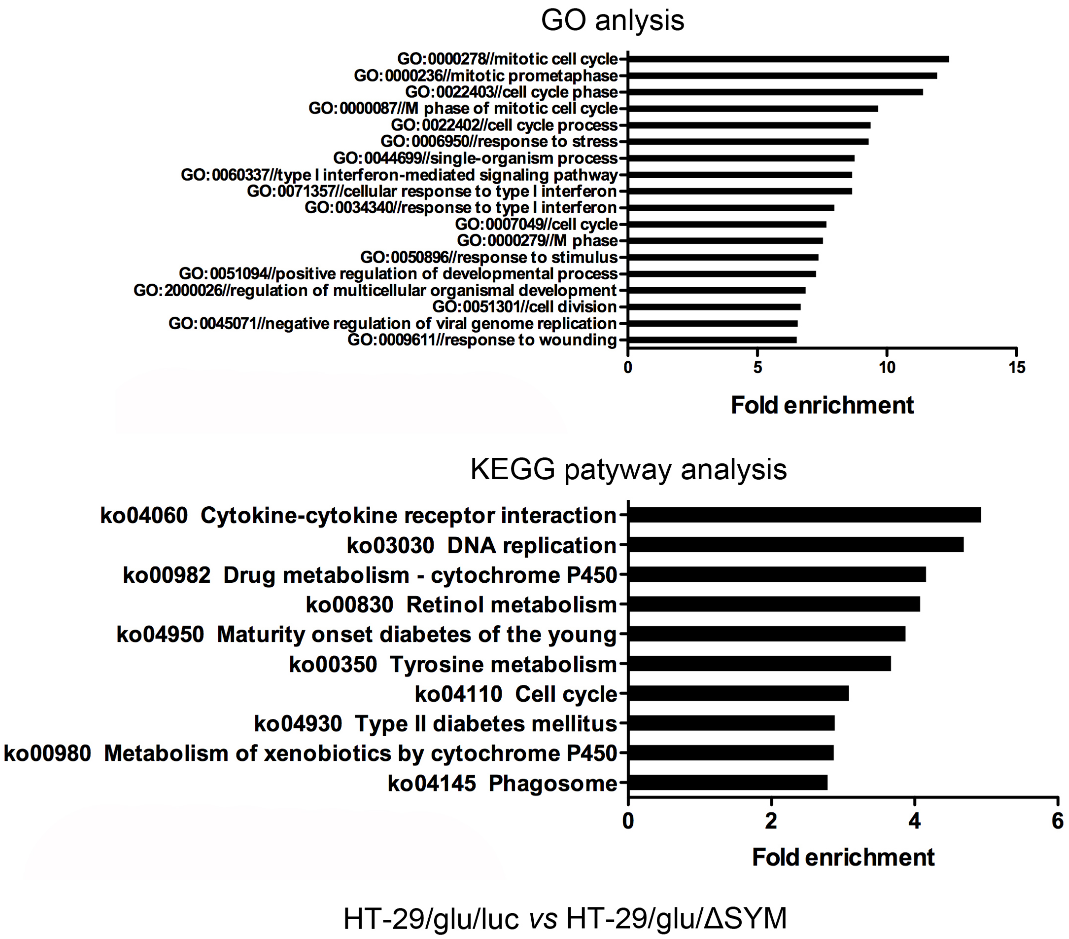

Figure S4. Co-IP analysis between YBX3 and symplekin mutants. FLAG tagged symplekin constructions were expressed in Caco-2 cells, Co-IP was performed using an anti-FLAG antibody. EV: empty vector; WT: wild type.

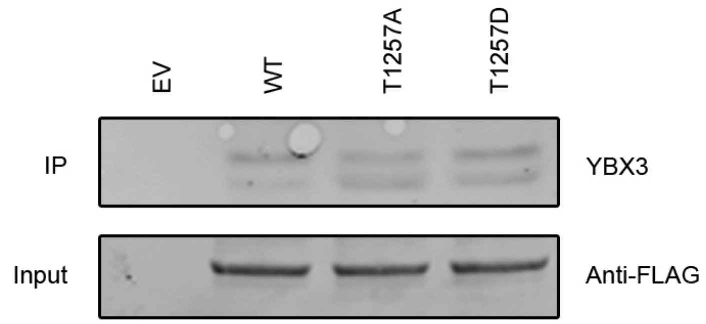

Table S1. Primers used in this study.

	Sequence 5'- 3'	Application
GAPDH Forward	TGTTGCCATCAATGACCCCTT	qRT-PCR
GAPDH Reverse	CTCCACGACGTACTCAGCG	qRT-PCR
Sucrase Forward	TGGAGGCCGTGGAATGACT	qRT-PCR
Sucrase Reverse	AACGATTGGGTGTCTGATTTTGA	qRT-PCR
ALPI Forward	TGAGGGTGTGGCTTACCAG	qRT-PCR
ALPI Reverse	GATGGACGTGTAGGCTTTGCT	qRT-PCR
CCND1 Forward	CCGTCCATGGGGAAGATC	qRT-PCR
CCND1 Reverse	ATGGCCAGCGGGAAGAC	qRT-PCR
PCNA Forward	CCTGCTGGGATATTAGCTCCA	qRT-PCR
PCNA Reverse	CAGCGGTAGGTGTCGAAGC	qRT-PCR
CDK1 Forward	AAACTACAGGTCAAGTGGTAGCC	qRT-PCR

CDK1 Reverse	TCCTGCATAAGCACATCCTGA	qRT-PCR
CCNA2 Forward	CGCTGGCGGTACTGAAGTC	qRT-PCR
CCNA2 Reverse	GAGGAACGGTGACATGCTCAT	qRT-PCR
CCNB1 Forward	AATAAGGCGAAGATCAACATGGC	qRT-PCR
CCNB1 Reverse	TTTGTTACCAATGTCCCCAAGAG	qRT-PCR
CCNB2 Forward	CCGACGGTGTCCAGTGATTT	qRT-PCR
CCNB2 Reverse	TGTTGTTTTGGTGGGTTGAACT	qRT-PCR
CCNE1 Forward	AAGGAGCGGGACACCATGA	qRT-PCR
CCNE1 Reverse	ACGGTCACGTTTGCCTTCC	qRT-PCR
CCNE2 Forward	TCAAGACGAAGTAGCCGTTTAC	qRT-PCR
CCNE2 Reverse	TGACATCCTGGGTAGTTTTCCTC	qRT-PCR
E2F1 Forward	CATCCCAGGAGGTCACTTCTG	qRT-PCR
E2F1 Reverse	GACAACAGCGGTTCTTGCTC	qRT-PCR
E2F2 Forward	CGTCCCTGAGTTCCCAACC	qRT-PCR
E2F2 Reverse	GCGAAGTGTCATACCGAGTCTT	qRT-PCR
CDC7 Forward	GAGGCGTCTTTGGGGATTCAG	qRT-PCR
CDC7 Reverse	GGTCCTACTTGTAACTGTGCTG	qRT-PCR
CDC25A Forward	GTGAAGGCGCTATTTGGCG	qRT-PCR
CDC25A Reverse	TGGTTGCTCATAATCACTGCC	qRT-PCR
CDC25C Forward	TCTACGGAACTCTTCTCATCCAC	qRT-PCR
CDC25C Reverse	TCCAGGAGCAGGTTTAACATTTT	qRT-PCR
PLK1 Forward	CCTGCACCGAAACCGAGTTAT	qRT-PCR
PLK1 Reverse	CCGTCATATTCGACTTTGGTTGC	qRT-PCR

MCM6 Forward	GAGGAACTGATTCGTCCTGAGA	qRT-PCR
MCM6 Reverse	CAAGGCCCGACACAGGTAAG	qRT-PCR
CCND1 Forward	TGCACCAAAGAGACAGAACCT	PCR/qPCR (ChIP)
CCND1 Reverse	TGAATTCGTGAGCGTGAGGG	PCR/qPCR (ChIP)
CDK1 Forward	TTCTCAGCCGCCCTTTCCT	PCR (ChIP)
CDK1 Reverse	AGCCAGCTTTGAAGCCAAGT	PCR (ChIP)
CCNB1 Forward	CCGTAGAAATGGAAAGTGTGCAA	PCR/qPCR (ChIP)
CCNB1 Reverse	CCTGGAAAGGTTAGCCGGG	PCR/qPCR (ChIP)
CCNA2 Forward	AGTTTGTTTCTCCCTCCTGCC	PCR/qPCR (ChIP)
CCNA2 Reverse	CTTGCAGTTCAAGTATCCCGC	PCR/qPCR (ChIP)
CCNE2 Forward	TCCGGAGGTGTCAGTCTGAGG	PCR (ChIP)
CCNE2 Reverse	CCGCCTGTGGTAAGTGATCG	PCR (ChIP)
E2F1 Forward	TATGTTCCGGTGTCCCCACG	PCR (ChIP)
E2F1 Reverse	CGGCGGCGGTTCCTATT	PCR (ChIP)
E2F2 Forward	TTTTGAATGAGGCAGAGAGGCT	PCR (ChIP)
E2F2 Reverse	AGCTCTGGAGCCCTTGTTTTT	PCR (ChIP)
CDC7 Forward	CTAGATGCCACCTTCCTGGTC	PCR (ChIP)
CDC7 Reverse	GAGCCCTTGGCTATGAGGTA	PCR (ChIP)
CDC25A Forward	CCTTCTGAGAGCCGATGACC	PCR (ChIP)
CDC25A Reverse	GTAATAGCGGCTCAGTGGGG	PCR (ChIP)
CDC25C Forward	ACATCACTAGTAAGGCGCGG	PCR (ChIP)
CDC25C Reverse	CCCATAGCCAGAGCAGGAT	PCR (ChIP)
MCM6 Forward	GCAAAGGCAGATGGGCTTTC	PCR (ChIP)

MCM6 Reverse	CCGGAACCAATCGTGACACA	PCR (ChIP)
ErbB2 Forward	CCTTAGGGGCTTCACTCACA	PCR (ChIP)
ErbB2 Reverse	GGGCGCTCTGAATCACTTCC	PCR (ChIP)
